# Sex-dependent differences in the secretome of human endothelial cells

**DOI:** 10.1186/s13293-020-00350-3

**Published:** 2021-01-07

**Authors:** Maria Grazia Cattaneo, Cristina Banfi, Maura Brioschi, Donatella Lattuada, Lucia M. Vicentini

**Affiliations:** 1 Dept of Medical Biotechnology and Translational Medicine, Università degli Studi di Milano, Via Vanvitelli 32, Milan, Italy; 2 Centro Cardiologico Monzino, IRCCS, Milan, Italy

**Keywords:** Secretome, Cellular stress, Apoptosis, Endothelial cells, Human, Pentraxin 3

## Abstract

**Background:**

Cellular sex has rarely been considered as a biological variable in preclinical research, even when the pathogenesis of diseases with predictable sex differences is studied. In this perspective, proteomics, and “omics” approaches in general, can provide powerful tools to obtain comprehensive cellular maps, thus favoring the discovery of still unknown sex-biased physio-pathological mechanisms.

**Methods:**

We performed proteomic and Gene Ontology (GO) analyses of the secretome from human serum-deprived male and female endothelial cells (ECs) followed by ELISA validation. Apoptosis was detected by FACS and Western blot techniques and efferocytosis through the ability of the macrophage cell line RAW 264.7 to engulf apoptotic ECs. PTX3 mRNA levels were measured by RT-qPCR.

**Results:**

Proteomic and GO analyses of the secretome from starved human male and female ECs demonstrated a significant enrichment in proteins related to cellular responses to stress and to the regulation of apoptosis in the secretome of male ECs. Accordingly, a higher percentage of male ECs underwent apoptosis in response to serum deprivation in comparison with female ECs. Among the secreted proteins, we reliably found higher levels of PTX3 in the male EC secretome. The silencing of PTX3 suggested that male ECs were dependent on its expression to properly carry out the efferocytotic process. At variance, female EC efferocytosis seemed to be independent on PTX3 expression.

**Conclusions:**

Our results demonstrated that serum-starved male and female ECs possess different secretory phenotypes that might take part in the sex-biased response to cellular stress. We identified PTX3 as a crucial player in the male-specific endothelial response to an apoptotic trigger. This novel and sex-related role for secreted proteins, and mainly for PTX3, may open the way to the discovery of still unknown sex-specific mechanisms and pharmacological targets for the prevention and treatment of endothelial dysfunction at the onset of atherosclerosis and cardiovascular disease.

**Supplementary Information:**

The online version contains supplementary material available at 10.1186/s13293-020-00350-3.

## Background

Almost all of our knowledge about fundamental biological processes has been provided by primary and established cell lines. However, most of these studies disregarded the sex origin of cells, and sex has rarely been considered as a relevant biological variable in preclinical research [1, 2]. This asexual approach has limited the understanding of potential sex-based differences not only in basic biological functions, but especially in pathophysiological mechanisms. This loss of knowledge is particularly restrictive when the pathogenesis of diseases showing predictable sex differences is studied [3, 4].

Atherosclerosis and cardiovascular disease (CVD) are classical examples of diseases where sex/gender differences have been described [5]. A significant body of evidence suggests that CVD is less prevalent in women than in men until midlife, and the female advantage in younger women has been attributed to estrogens, which are lost with menopause. Since the earliest event in the onset of atherosclerosis and CVD is endothelial dysfunction, many studies have been focused on endothelium and endothelial cells (ECs). Although evidence of important sex/gender differences in the endothelial functions has been described in both rodents and humans [6, 7], the sex of cells was not consistently reported in studies involving ECs, even when the effects of sex hormones were analyzed [8–10]. However, when primary cultures of male and female ECs from different vascular beds were independently studied, some inborn sex differences appeared [11–18].

In this context, proteomics, and “omics” approaches in general, can provide powerful tools to obtain detailed cellular maps at the molecular level, thus favoring the comprehension of the molecular basis of pathogenesis. Sex/gender-specific differences at the proteomic level throughout non-sexual organs and tissues have been described [19]. Such differences have been reported also for dioicous plants and many animals in all phyla [19]. However, molecular data for human tissues and primary cells are still scarce, as well as proteomic studies focused on sex-related differences in ECs [17, 20]. Likewise, very few proteomics studies have analyzed the EC secretome [21–23] that consists of all the proteins released by vascular cells and that controls a plethora of biological processes, thus representing a potential source for biomarkers and therapeutic target discoveries.

In this study, we have performed for the first time a proteomic analysis of the secretome from human male and female ECs isolated from the umbilical cords (human umbilical vein ECs (HUVECs)). Among others, we consistently found higher levels of the long pentraxin 3 (PTX3), a highly conserved member of the pentraxin family [24], in the secretome of male ECs. Therefore, we focused our attention on the biological meaning of the observed sex-dimorphic production of PTX3, by evaluating its role in the response of ECs to an external source of cellular stress represented by serum deprivation.

## Methods

### Cell cultures

HUVECs were freshly isolated from anonymized umbilical cords from healthy male or female babies born at term from healthy mothers—free of drugs and of hepatitis or human immunodeficiency virus (HIV) infection—essentially as previously described [25]. Cells were pooled from two different umbilical cords—to minimize variability associated with cells derived from a single male or female newborn donor—and used at passages 1–5. HUVECs were routinely grown in 199 medium supplemented with 20% fetal bovine serum (FBS), 25 μg/ml endothelial cell growth supplement (ECGS), and 50 μg/ml heparin on 0.1% gelatin-coated surfaces. All the experiments were performed on ECs or conditioned media collected after an overnight incubation in the absence of serum or in the presence of 2% FBS, unless otherwise indicated.

### Label-free mass spectrometry analysis

Secretome samples for proteomic analysis were desalted, concentrated, and digested as previously described [23]. After lyophilization, the secreted protein pellets were dissolved in 25 mmol/l NH_4_HCO_3_ containing 0.1% RapiGest (Waters Corporation), sonicated, and centrifuged at 13,000*g* for 10 min. Samples were then incubated 15 min at 80 °C and reduced with 5 mmol/l DTT at 60 °C for 15 min, followed by carbamidomethylation with 10 mmol/l iodoacetamide for 30 min at room temperature in the darkness. Then, sequencing grade trypsin (Promega) was added to each sample (1 μg every 50 μg of proteins) and incubated overnight at 37 °C. After digestion, 2% TFA was added to hydrolyze RapiGest and inactivate trypsin. Tryptic peptides were used for label-free mass spectrometry analysis (LC-MS^E^) performed on a hybrid quadrupole time of flight mass spectrometer coupled with a nanoUPLC system and equipped with a Trizaic source (Waters Corporation) as previously described in detail [23, 26]. Statistical analysis has been performed by means of Progenesis QIP v 4.1 (Nonlinear Dynamics) using a UniProt human protein sequence database (v2017-1). The mass spectrometry proteomics data have been deposited to the ProteomeXchange Consortium via the PRIDE [27] partner repository with the dataset identifiers PXD020375 and 10.6019/PXD020375.

### Gene Ontology analysis

Data were analyzed with the Search Tool for the Retrieval of Interacting Genes/Proteins (STRING 11.0) database [28] as previously described [29] to identify enriched Gene Ontology (GO) terms in the biological process, molecular function, or cellular component categories. We specifically employed the enrichment widget of STRING, which calculates an enrichment *p* value based on the hypergeometric test and the method of Benjamini and Hochberg for correction of multiple testing (*p* value cutoff of < 0.05).

### Measurement of secreted PTX3

Conditioned media (2.0–2.5 ml/flask) were collected after an overnight incubation from confluent HUVEC monolayers cultured in 25-cm^2^ gelatin-coated flasks. After a 10-min centrifugation (300*g* at 4 °C) to remove cellular debris, media were aliquoted and stored at − 80 °C. The concentration of PTX3 was measured using the Human Pentraxin 3/TSG-14 Immunoassay (R&D Systems, Inc.).

### Cell images

Photographs of HUVECs were acquired at a × 10 magnification with an Olympus U-CMAD3 phase-contrast microscope equipped with an Olympus digital camera.

### Measurement of cell number

The number of cells was measured by crystal violet on HUVECs plated at a density of 2.0 × 10^4^ cells/well in 0.1% gelatin-coated 96-well microplates the day before the experiment. Crystal violet binds to DNA and proteins, thus allowing the detection of adherent cells [30]. Briefly, cells were fixed with ice-cold 100% methanol for 10 min and stained with the crystal violet (0.5% in 20% methanol) for about 15–20 min. After multiple washes with deionized water, the plate was air-dried, and the crystal violet stain was solubilized in DMSO (100 μl/well) before the measurement of optical density at 595 nm by a multiplate reader (Victor™, PerkinElmer).

### Measurement of reactive oxygen species

Reactive oxygen species (ROS) were detected as previously described [31] on HUVECs plated at a density of 2.0 × 10^4^ cells/well in 0.1% gelatin-coated black 96-well microplates the day before the experiment. Cells were loaded for 30 min at 37 °C in the dark with the fluorescent dye 5(6)-carboxy-2′7′-dichlorofluorescein diacetate (CM-DCFDA, 10 μM) in HBSS buffer (Hepes 25 mM pH 7.4, NaCl 120 mM, KCl 5.4 mM, CaCl_2_ 1.8 mM, NaHCO_3_ 25 mM, glucose 15 mM) containing 1% FBS. Afterwards, the cells were washed, and fluorescence was assessed after 30 min by a multiplate reader (Victor™, PerkinElmer) with excitation and emission wavelengths of 485 and 530 nm, respectively. The protein content of each well was quantified by the BCA assay (Pierce) to normalize sample-to-sample variation.

### Apoptosis assays

Quantification of apoptosis/necrosis was performed by Annexin V-FITC conjugate and propidium iodide (PI) staining (Immunological Sciences) followed by fluorescence-activated cell sorting (FACS) performed with a FACScalibur flow cytometer equipped with a 488-nm argon laser (Becton Dickinson). The collected data were evaluated by the Cell Quest software. In addition, active caspase-3 and PARP-1 proteins were detected by conventional Western blot on total cell lysates prepared in SDS-PAGE sample buffer (62 mM Tris-HCl pH 6.8, 2% sodium dodecyl sulfate (SDS), 10% glycerol, 5% 2-mercaptoethanol, and 0.04% bromophenol blue). Densitometric analyses of immunoblots were performed using the National Institute of Health (NIH) ImageJ software package. Full-length unedited blots are shown in Additional file 4.

### Small interfering RNA transfection

To silence PTX3 expression, HUVECs were transfected with the Hs_PTX3_1 FlexiTube siRNA duplexes against human PTX3 (SI00695947, Qiagen). An AllStars Negative Control FlexiTube siRNA (Qiagen) was used as a control. Both siRNAs were individually transfected at a 5-nM concentration using the PepMute transfection reagent according to the manufacturer’s instructions (Signa Gen Laboratories). For the in vitro efferocytosis and FACS analysis shown in Fig. 5e and f, respectively, and for the adhesion assay in Supplementary Fig. 1, HUVECs were transfected 48 h before calcein-AM loading and overnight serum deprivation. The knockdown of PTX3 expression was analyzed by reverse transcription and quantitative real-time PCR (RT-qPCR) and by ELISA measurement of the secreted PTX3 protein.

### Total RNA extraction and RT-qPCR

Total cellular RNA was extracted with the Total RNA Purification Kit (Norgen Biotek Corp) and reverse transcribed (1 μg) as previously described [32]. RT-qPCR was performed in triplicate with 2.5 μl of cDNA incubated in 22.5 μl IQ Supermix containing primers and SYBRGreen fluorescence dye (Bio-Rad Laboratories) using the iCycler Optical System (Bio-Rad Laboratories). The sequences of PTX3 primers were as follows: forward, 5′-TGC GAT TCT GTT TTG TGC TC-3′; reverse, 5′-TGA AGA GCT TGT CCC ATT CC-3′. GAPDH was used as a normalizer. The sequences of GAPDH primers were as follows: forward, 5′-ACG GAT TTG GTC GTA TTG GGC-3′; reverse, 5′-CTC CTG GAA GAT GGT GAT GG-3′.

### Efferocytosis and adhesion assays

RAW 264.7 macrophages were plated at a density of 5.0 × 10^4^ cells/well in 100 μl/well of DMEM + 5% FBS in a black 96-well microplate. HUVECs were loaded for 30 min with calcein-AM (2 μM) in HBSS before overnight incubation in a 2% FBS-containing medium. After detachment and re-suspension in the conditioned media, HUVECs were collected by centrifugation and overlaid on the RAW 264.7 cells at a 1:1 ratio of macrophages:HUVECs in a 100-μl volume of 20% FBS-containing 199 medium [33]. A 100-μl aliquot of HUVEC suspension was concurrently plated in empty wells to measure the total fluorescence added to macrophages. Following 2 h of incubation, cells were washed 3 times with HBBS—to remove cells that were not engulfed—and the RAW 264.7-associated fluorescence was assessed by a multiplate reader (Victor™, Perkin Elmer). Data are expressed as percent (%) of engulfed cells that is the RAW 264.7 cell-associated fluorescence divided by the total fluorescence multiplied by 100*.*

In some experiments, calcein-loaded HUVECs were also plated in a black 96-well microplate coated with 0.1% gelatin. After 2 h of incubation at 37 °C, cells were washed as described above, and the well-associated fluorescence (related to the number of adherent cells) was measured by a multiplate reader. Data are expressed as percent (%) of adherent cells that is the well-associated fluorescence divided by the total fluorescence multiplied by 100.

### Statistical procedures

Unless otherwise indicated, data are expressed as mean ± s.e.m. of *n* independent experiments performed on different cell preparations. Statistical analysis was carried out using unpaired Student’s *t* test or 2-way analysis of variance (ANOVA) followed by Sidak’s multiple comparison test. In the 2-way ANOVA analyses, we considered as a row factor the sex of cells and as a column factor the treatment (20% vs 2% FBS in Figs. 2d, e and 3a, b, and ctrl vs PTX3 siRNA in Fig. 5c–e). *p* values of < 0.05 were considered significant. All the analyses were performed using the GraphPad Prism software (version 8.4.2).

### Reagents and antibodies

All tissue culture reagents were from Euroclone, except ECGS and heparin (Sigma Aldrich). Crystal violet was from Sigma Aldrich and CM-DCFDA and calcein-AM from Cayman Chemicals. Primary antibodies used were mouse monoclonals anti-caspase-3 (Enzo Life Sciences, ALX-804-305) and anti-actin (BD Biosciences, 612656) and rabbit monoclonal anti-cleaved PARP-1 (Cell Signaling, #5625). Horseradish peroxidase (HRP)-conjugated secondary antibodies were from Dako (#P0260 and #P0399, for rabbit anti-mouse and swine anti-rabbit antibodies, respectively). Proteins in Western blot were detected by the LiteAblot Turbo Extra-Sensitive Chemiluminescent Substrate (Euroclone).

## Results

### Proteomic analysis of male and female EC secretome

**Fig. 1:**
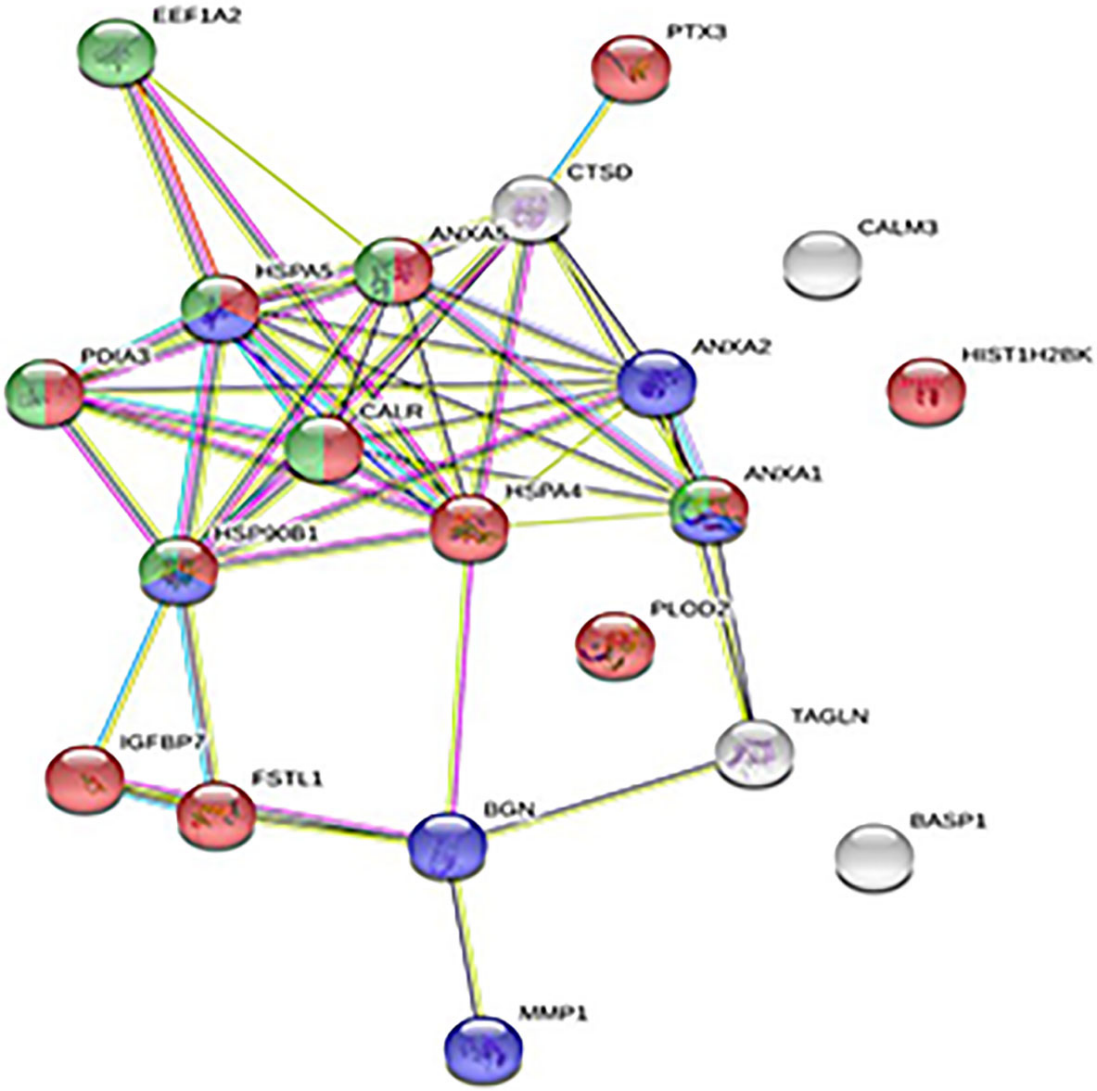
Gene Ontology analysis of the proteins more secreted by male ECs (see Table 1). String network generated with the proteins more abundant in the male EC secretome highlighting the enriched biological processes: red, cellular response to stress; blue, cellular response to cytokine stimulus; green, regulation of the apoptotic process

To evaluate still unknown differences, in terms of protein secretion, between human male and female HUVECs (abbreviated as ECs), we analyzed their secretome after an overnight incubation in the absence of serum by means of a mass spectrometry-based proteomic approach, LC-MS^E^. Label-free LC-MS^E^ analysis allowed us to identify and quantify EC-secreted proteins (dataset PDX020375). Among these proteins, we identified 20 proteins significantly more abundant in the secretome of male ECs and 3 proteins more present in the female secretome (Table 1). Of note, a Gene Ontology (GO) analysis, performed with STRING, on the panel of proteins increased in the male secretome, showed significant enrichment in the biological process categories of the GO terms related to responses to stress (*p* = 0.0029), to cytokine stimulus (*p* = 0.01), and to the regulation of apoptosis (*p* = 0.014) (Fig. 1 and Supplementary Table 1, see Additional file 1). Conversely, proteins involved in metabolic processes, such as the carbohydrate derivative and glyceraldehyde-3-phosphate pathways, were identified by GO analysis in the female EC secretome (Supplementary Table 2, see Additional file 2). These results suggest that male and female ECs show different secretory phenotypes, due to the secretion of different sets of proteins, in response to serum deprivation.

**Table 1:** List of the proteins differentially expressed in the secretome of female and male ECs identified by LC-MS^E^

Accession	Description	Peptide count/unique peptides	Confidence score	ANOVA (***p***)	Max. fold change
**Higher secretion by female cells**					
P00352	Retinal dehydrogenase 1, ALDH1A1	9/9	66.77	5.83E−09	1.80
P52209	6-Phosphogluconate dehydrogenase decarboxylating, PGD	3/3	18.43	3.74E−06	1.39
P55072	Transitional endoplasmic reticulum ATPase, VCP	17/17	120.47	8.45E−07	1.31
**Higher secretion by male cells**					
O00469	Procollagen-lysine_2-oxoglutarate 5-dioxygenase 2, PLOD2	3/3	16.84	1.24E−05	1.58
O60814	Histone H2B type 1-K, HIST1H2BK	3/3	21.68	1.20E−06	1.32
P03956	Interstitial collagenase MMP1	15/15	114.51	1.41E−08	2.84
P04083	Annexin A1, ANXA1	6/6	39.36	1.95E−06	1.25
P07339	Cathepsin D, CTSD	3/3	18.87	6.45E−05	1.34
P07355	Annexin A2, ANXA2	17/17	137.10	2.19E−06	1.25
P08758	Annexin A5, ANXA5	6/6	40.18	1.08E−06	1.41
P11021	78 kDa glucose-regulated protein, HSPA5	11/9	75.79	2.72E−06	1.20
P14625	Endoplasmin, HSP90B1	6/5	36.50	3.11E−05	1.25
P21810	Biglycan, BGN	13/13	86.33	2.20E−07	1.67
P26022	Pentraxin-related protein PTX3	9/9	67.95	4.35E−06	2.32
P27797	Calreticulin, CALR	8/8	50.99	5.28E−05	1.31
P30101	Protein disulfide-isomerase A3, PDIA3	6/6	38.64	2.88E−05	1.32
P34932	Heat shock 70 kDa protein 4, HSPA4	2/2	10.64	0.000344	1.20
P62158	Calmodulin CALM3	5/5	51.14	2.62E−08	1.30
P80723	Brain acid soluble protein 1, BASP1	2/2	11.70	2.48E−06	1.48
Q01995	Transgelin, TAGLN	2/2	12.26	0.000132	1.39
Q05639	Elongation factor 1-alpha 2, EEF1A2	6/2	36.12	0.002882	1.36
Q12841	Follistatin-related protein 1, FSTL1	3/3	17.39	1.53E−05	1.26
Q16270	Insulin-like growth factor-binding protein 7, IGFBP7	12/12	96.80	1.43E−07	1.37

### PTX3 is more secreted by male ECs in comparison with female ECs

**Fig. 2:**
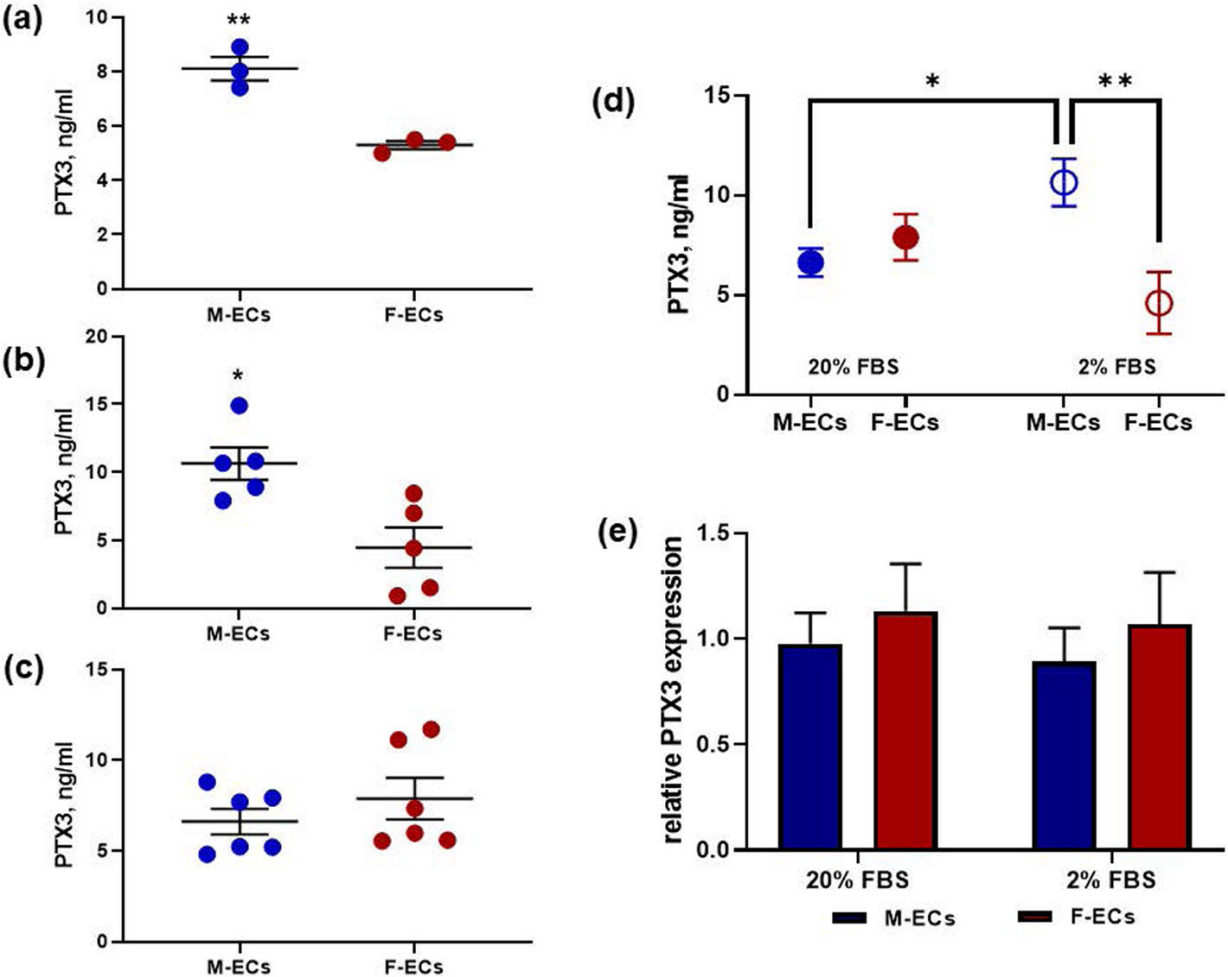
PTX3 is more secreted by male ECs in comparison with female ECs. The levels of PTX3 were measured by an immune-enzymatic assay on conditioned media collected from male ECs (M-ECs, blue dots) or female ECs (F-ECs, deep red dots) after an overnight incubation in **a** serum-free media, **p < 0.01, *n* = 3; **b** 2% FBS-containing media, *p < 0.05, *n* = 5; and **c** 20% FBS-containing media, *n* = 6. *N* is the number of independent experiments, and lines and error bars show the mean ± s.e.m. of these experiments. Individual dots are the mean of duplicate samples from each experiment. In **d**, the PTX3 levels shown in **b** (2% FBS, open dots) and **c** (20% FBS, closed dots) were summarized and analyzed by a 2-way ANOVA followed by Sidak’s multiple comparison test, *p < 0.05, **p < 0.01; *n* = 5–6 for 2% and 20% FBS, respectively. Interaction between sexes is significant, **p < 0.01. **e** PTX3 mRNA was measured by RT-qPCR and normalized to the housekeeping gene GAPDH in M-ECs (blue bar) and F-ECs (deep red bar) collected after an overnight incubation in 20% or 2% FBS as indicated. No significant differences between the groups; 2-way ANOVA followed by Sidak’s multiple comparison test, *n* = 4

Among the proteins differentially secreted by male and female ECs, we focused our interest on PTX3, for two main reasons: (i) a role for PTX3 has been proposed in the response to vascular damage and in the development and progression of atherosclerosis [34, 35], and (ii) we and others have previously shown that PTX3 is one of the more represented proteins in the secretome of human ECs, when cells are studied without sex segregation [21–23]. Despite these suggestions, the biological significance of the secreted PTX3 in the human EC pathophysiology is still unknown, as well as the existence of a possible sex dimorphism in its production and/or biological role. The presence of PTX3 in the conditioned media obtained from male or female ECs, incubated overnight in serum-free or in 2% FBS-containing media, was validated by a quantitative immune-enzymatic assay. As shown in Fig. 2, the levels of PTX3 were significantly higher in male ECs in comparison with female ECs either in the absence of serum (Fig. 2a, 8.1 ± 0.4 vs 5.3 ± 0.1 ng/ml for male and female ECs, respectively) or in the presence of 2% FBS (Fig. 2b, 10.6 ± 1.2 vs 4.5 ± 1.5 ng/ml for male and female ECs, respectively). The difference in the levels of secreted PTX3 was still conserved when male/female ECs were incubated overnight in 0.5% bovine serum albumin-containing medium (data not shown). At variance, no significant differences between male and female ECs were observed when PTX3 was measured in media from cells incubated overnight in standard conditions, i.e., 20% FBS (Fig. 2c). Notably, when control- and serum-deprived conditioned media were compared, the amount of secreted PTX3 was increased only in male ECs (Fig. 2d). No significant differences were observed between control and starved female ECs, where a tendency to a decrease rather than an increase in the PTX3 levels was observed (Fig. 2d). Finally, the constitutive expression of PTX3 measured by RT-qPCR did not differ between male and female ECs either in 20% or 2% FBS (Fig. 2e). Collectively, these results reveal different amounts of PTX3 in the secretome of starved male and female ECs, thus suggesting a possible sex-dependent function for secreted PTX3 in the endothelial response to serum deprivation.

### Serum starvation acts as a stressor in male and female ECs

**Fig. 3:**
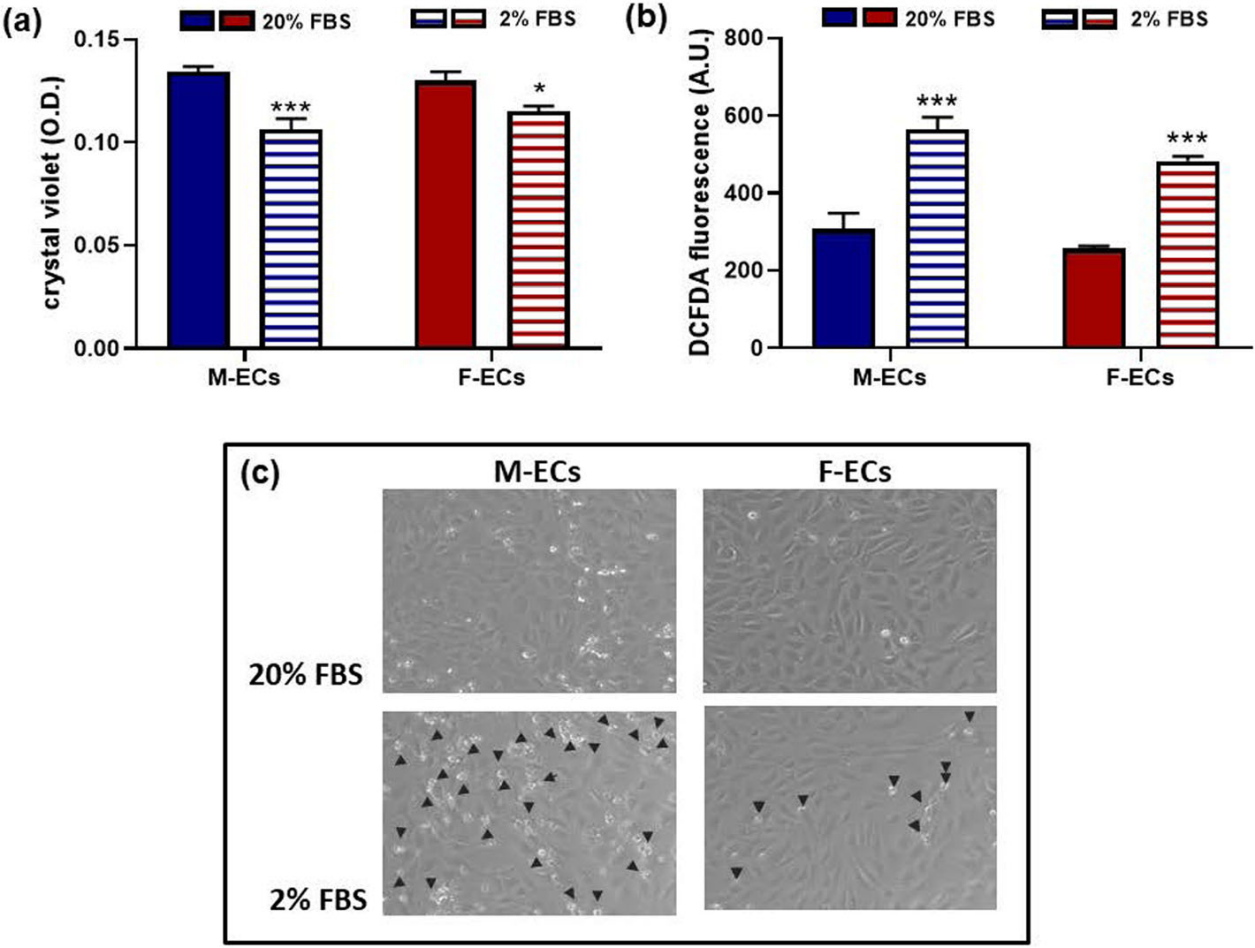
Serum starvation acts as a stressor in male and female ECs. **a** Adherent M-ECs (blue bars) and F-ECs (deep red bars) were measured after an overnight incubation in 20% FBS (solid bars) or 2% FBS (horizontal bars). Data are expressed as crystal violet absorbance (O.D.). ***p < 0.001, *p < 0.05 vs 20% FBS in M-ECs and F-ECs, respectively; 2-way ANOVA followed by Sidak’s multiple comparison test, *n* = 3. No significant interaction between sexes. **b** Cell-associated fluorescence was measured in cells treated as in **a**. Data are expressed as DCFDA fluorescence (A.U.) normalized to the well protein content. ***p < 0.001 vs 20% FBS; 2-way ANOVA followed by Sidak’s multiple comparison test, *n* = 3. No significant interaction between sexes. **c** Representative images of M-EC and F-EC monolayers (left and right panels, respectively) after an overnight incubation in 20% FBS (upper panels) or 2% FBS (lower panels). Arrows indicate cellular debris

Serum starvation represents an important stress factor, which deprives cells of crucial metabolites and growth factors, thus breaking the physiological cellular homeostasis. Consequently, cells can undergo morphological alterations, accumulate reactive oxygen species (ROS), stop growth, and eventually die. After an overnight incubation in 2% FBS-containing medium (i) the number of adherent ECs, evaluated by crystal violet staining, was significantly reduced in comparison with control cells, i.e., cells incubated in 20% FBS, in both sexes (by 20.7 ± 4.2 and 12.7 ± 3.0% for male and female ECs, respectively; *p* = 0.152, *n* = 6) (Fig. 3a); and (ii) the intracellular ROS content, measured with the fluorescent dye DCFDA, was increased by about 2-fold in both male and female ECs (Fig. 3b). Accordingly to our previous results, no significant differences in intracellular ROS and cell number were found between male and female ECs cultured in 20% FBS [14, 36]. Of note, phase contrast microscopy images of male and female EC monolayers incubated overnight in 2% FBS showed the occurrence of cellular debris, especially in male ECs (Fig. 3c). These results demonstrate that an overnight serum starvation adversely affects EC behavior.

### Serum starvation induces apoptosis in male ECs

**Fig. 4:**
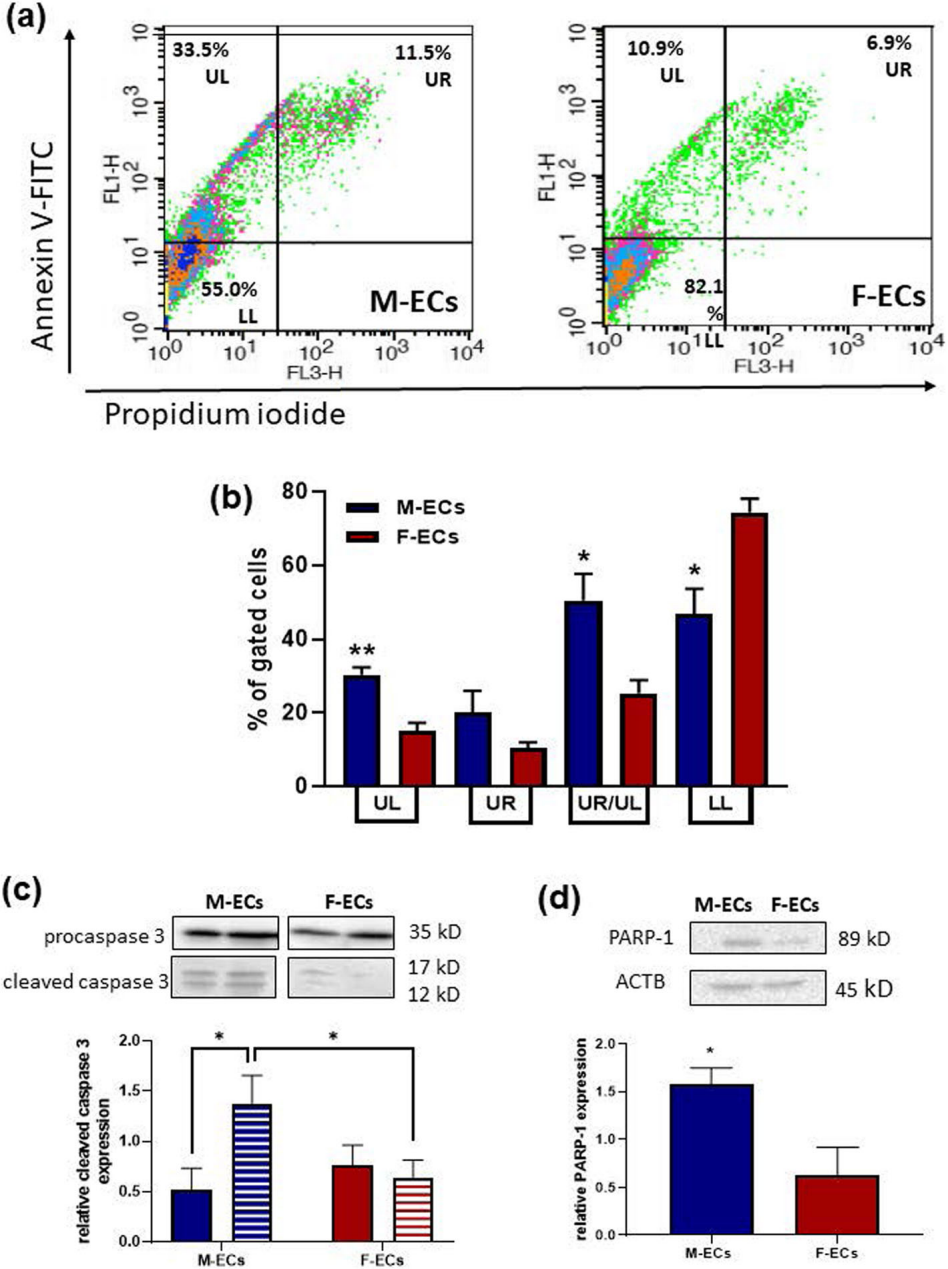
Serum starvation induces apoptosis in male ECs. **a** Representative flow cytometry graphs illustrating the percentage of early apoptotic cells (upper left quadrant (UL)), late apoptotic cells (upper right quadrant (UR)), and live cells (lower left quadrant (LL)) in serum-starved M-ECs and F-ECs (left and right panels, respectively). **b** Means ± s.e.m. of the percentage of gated M-ECs and F-ECs (blue and deep red bars, respectively) in the UL, UR, and LL quadrants from 4 to 5 independent experiments. *p < 0.05, **p < 0.01 vs F-ECs, *n* = 4–5. **c** Pro- and cleaved caspase-3 were evaluated by immunoblotting in M- and F-EC lysates (30 μg/lane) prepared after an overnight incubation in 2% FBS. Upper panel: a representative blot out of six. Lower panel: a densitometric analysis of the ratio between cleaved and pro-caspase-3 forms is shown. *p < 0.05, *n* = 6. **d** Cleaved PARP-1 was evaluated as in **c**. β-Actin was used as a loading control. Upper panel: a representative blot out of three. Lower panel: the ratio between cleaved PARP-1 and β-actin is shown. *p < 0.05, *n* = 3

Cells can adopt multiple strategies in response to external stressors, such as serum deprivation, ranging from the activation of pathways that promote survival to eliciting programmed cell death to remove damaged cells. In this context, a central role for PTX3 in the response to tissue damage and repair has been proposed [37]. Thus, to shed light on the defense mechanisms activated by ECs in response to serum starvation, and to elucidate potential sex differences in the role of PTX3 in the execution of these strategies, we analyzed the apoptotic process. To this aim, Annexin V-conjugated FITC and PI staining followed by FACS analysis were used to detect apoptotic cells in male and female ECs incubated overnight in a 2% FBS-containing medium. Starved male ECs showed higher percentages of both early and late apoptotic cells—sorted in the upper left (UL) and in the upper right (UR) quadrants, respectively—in comparison with female ECs (Fig. 4a), with the greater increase in the fraction of cells engaged in the early apoptotic process (30.4 ± 2.0 and 15.0 ± 2.3% for M-ECs and F-ECs, respectively) (Fig. 4b). Collectively, apoptotic cells (expressed as the sum of cells sorted in both the UL and UR quadrants) account for about 50% of the gated cells in male ECs, whereas only 25% of the cells were apoptotic in female ECs (Fig. 4b). No significant differences were observed between ECs of both sexes in the percentage of necrotic cells (lower right quadrant (LR)) (1.0 ± 0.6 and 0.6 ± 0.4% for M-ECs and F-ECs, respectively). The higher tendency of male ECs to undergo apoptosis was confirmed by Western blot analysis of serum-deprived male and female EC lysates. The 17- and 12-kD bands corresponding to the cleaved form of caspase-3—one of the key executioners of apoptosis—were significantly higher (by 3.44 ± 0.76-fold, *n* = 6) in lysates from starved male ECs in comparison with female ECs (Fig. 4c). Likewise, the 89-kD fragment of the cleaved poly (ADP-ribose) polymerase (PARP-1)—one of the main targets of activated caspase-3–was evident only in lysates from serum-starved male ECs (Fig. 4d). All these results unveil a different tendency of male and female ECs to undergo apoptosis in response to an external stressor represented by serum starvation.

### The expression of PTX3 is required for efferocytosis in male ECs

**Fig. 5:**
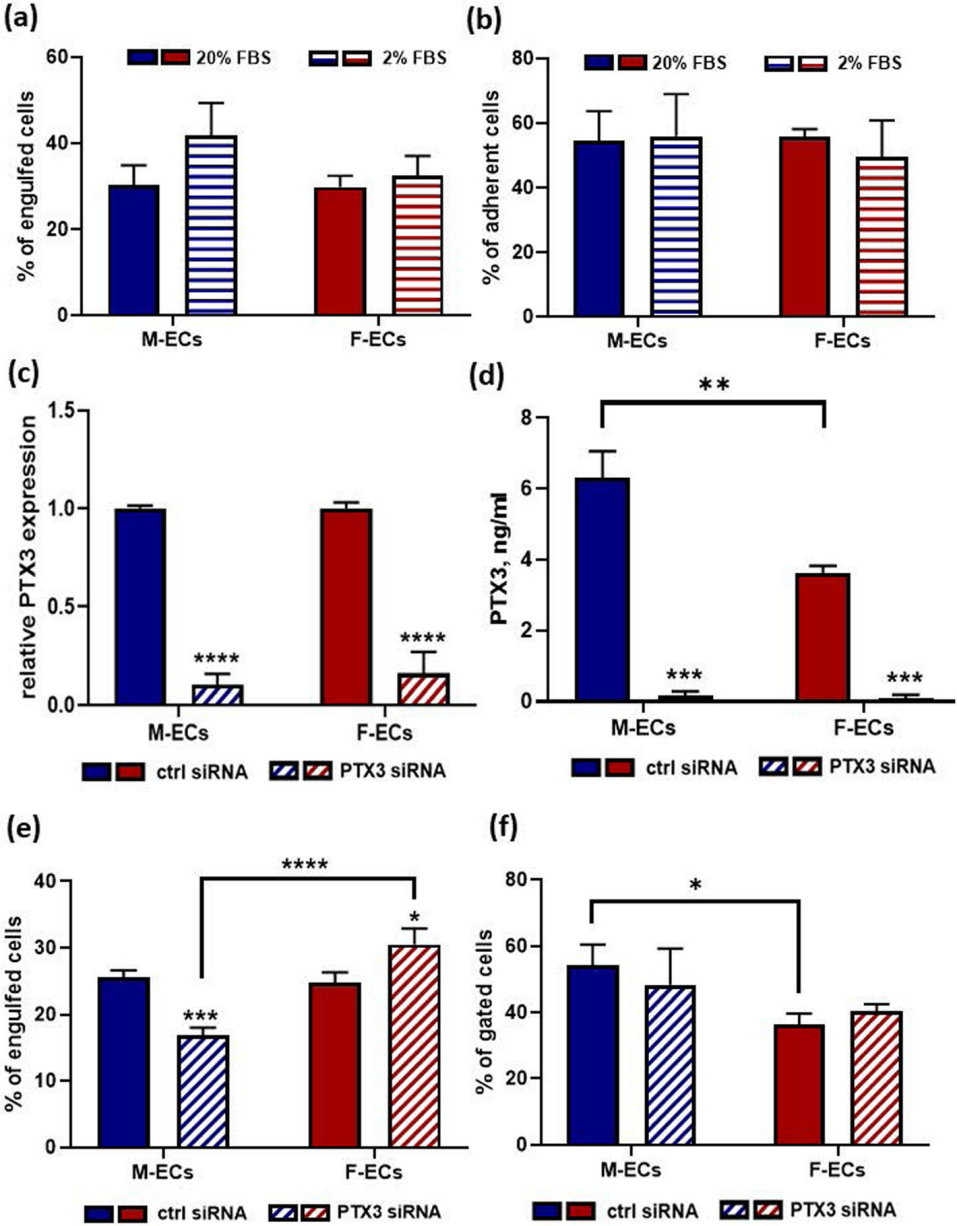
The expression of PTX3 is required for efferocytosis in male ECs. **a** Calcein-loaded M-ECs (blue bars) and F-ECs (deep red bars) were overlaid on a RAW 264.7 cell monolayer after an overnight incubation in 20% (solid bars) or 2% (horizontal bars) FBS. Two hours later, RAW-associated fluorescence was measured. Data are expressed as percent (%) of engulfed cells, that is the ratio between the RAW-associated fluorescence and the total added fluorescence (53,866 ± 9369 and 53,345 ± 7761 A.U. for 20%- and 2%-treated cells, respectively) multiplied by 100. No significant differences between the groups, *n* = 5/7 for 20% FBS-treated M-ECs and F-ECs, respectively; *n* = 10 for 2% FBS-starved M-ECs and F-ECs. **b** M-ECs (blue bars) and F-ECs (deep red bars) treated as described in **a** were plated on 0.1% gelatin-coated well, and 2 h later, well-associated fluorescence was measured. Data are expressed as percent (%) of adherent cells, that is the ratio between the well-associated fluorescence and the total added fluorescence (27,717 ± 3412 and 32,078 ± 4324 A.U. for 20%- and 2%-treated cells, respectively) multiplied by 100. No significant differences between the groups, *n* = 3 for 20% FBS-treated M-ECs and F-ECs; *n* = 5 for 2% FBS-starved M-ECs and F-ECs. **c**, **d** M-ECs (blue bars) and F-ECs (deep red bars) were transfected for 48 h with control (ctrl siRNA, solid bars) or PTX3 siRNA (diagonal bars). In **c**, PTX3 mRNA was measured by RT-qPCR and normalized to the housekeeping gene GAPDH. ****p < 0.0001 vs ctrl siRNA, *n* = 3. In **d**, secreted PTX3 protein was measured. ***p < 0.001 vs ctrl siRNA, **p < 0.01 between PTX3 levels in ctrl siRNA-transfected M-ECs and F-ECs, *n* = 2. Interaction between sexes is significant, *p < 0.05.  2-way ANOVA followed by Sidak’s multiple comparison test. **e** M-ECs (blue bars) and F-ECs (deep red bars) transfected with control (ctrl siRNA, solid bars) or PTX3 siRNAs (diagonal bars) were loaded with calcein-AM, and after an overnight serum deprivation, the percent of engulfed cells was measured as described in **a**. ***p < 0.001, *p < 0.05 vs ctrl siRNA in M-ECs and F-ECs, respectively; ****p < 0.0001 between PTX3 siRNA in M-ECs and F-ECs, *n* = 6. Interaction between sexes is significant, ***p < 0.001.  2-way ANOVA followed by Sidak’s multiple comparison test. **f** M-ECs (blue bars) and F-ECs (deep red bars) were transfected with control (ctrl siRNA, solid bars) or PTX3 (diagonal bars) siRNAs, and after an overnight serum deprivation, the percent of gated cells was measured by FACS as described in Fig. 4a, b. Data are expressed as the sum of the cells gated in the UL (early apoptotic) and UR (late apoptotic) quadrants. *p < 0.05 between ctrl siRNA-transfected M-ECs and F-ECs, *n* = 3

To properly conclude the apoptotic process, damaged cells need to be efficiently removed in vivo by tissue macrophages via efferocytosis [38, 39]. This process can be reproduced in vitro, and the number of apoptotic cells engulfed by a macrophage cell line can be quantified [33]. When calcein-loaded ECs exposed overnight to 2% FBS were incubated with a monolayer of RAW 264.7 cells, the engulfment of male ECs was slightly increased (by about 30%) in comparison with female ECs (41.9 ± 7.5% vs 32.5 ± 4.6%, respectively) (Fig. 5a). Although small and not significant, this disparity was lost in cells cultured in 20% FBS where an equal fraction of male and female ECs was engulfed (30.4 ± 0.5% and 29.9 ± 2.6%, respectively) (Fig. 5a). Notably, the engulfment of M-ECs was increased (by about 38%) as a consequence of serum deprivation (41.9 ± 7.5% vs 30.4 ± 0.5%, respectively), whereas it was unaffected in starved female ECs (29.9 ± 2.6% vs 32.5 ± 4.6% in 20% and 2% FBS, respectively). In addition, differences in engulfed cells did not depend on distinct attachment properties endowed by male and female ECs (Fig. 5b). A role for PTX3 in the clearance of apoptotic neutrophils and in their uptake by macrophages has been proposed [40]. Thus, we silenced PTX3 to reveal its putative involvement in in vitro male and female EC efferocytosis. The ability of siRNA (5 nm for 48 h) to equally and efficiently knockdown PTX3 mRNA and secreted protein in male and female ECs is shown in Fig. 5c, d. Outstandingly, the percentage of phagocytized male ECs was decreased by about 35% in silenced cells, whereas the engulfment of female ECs was slightly increased (by about 20%) in the absence of PTX3 expression (Fig. 5e). Notably, PTX3 silencing did not affect the metabolic, proliferative, and attachment properties of male and female ECs of its own (Supplementary Fig. 1, see Additional file 3). Most importantly, PTX3 silencing did not modify the tendency of male ECs to undergo apoptosis (Fig. 5f). Therefore, male ECs appear to be partially dependent on the expression of PTX3 to properly carry out the efferocytotic process. At variance, female EC efferocytosis seems to be independent of PTX3 expression.

## Discussion

Proteomic studies on the non-cellular fractions of ECs are so far very limited [21–23], and data on male and female endothelial secretome have not yet been available. In this study, we performed for the first time a proteomic analysis of the secretome from human serum-deprived male and female ECs, showing a significant enrichment in proteins related to cellular responses to stress and to the regulation of apoptosis in the secretome of male ECs. Consistently, a significantly higher percentage of male ECs underwent apoptosis in comparison with female ECs, when exposed to serum starvation as environmental stress. Among the secreted proteins, we reliably found higher levels of PTX3 in the male EC secretome. The knockdown of PTX3 expression revealed its requirement for the proper execution of efferocytosis—that is, the final step of apoptosis in which damaged cells are recruited and removed by macrophages—only in male ECs, but not in female ECs. Taken together, these data suggest a novel and sex-related role for secreted PTX3 in the pathophysiology of human ECs.

Notably, among the differentially abundant secreted proteins, biglycan (BGN) is the only protein encoded by a gene located in sexual chromosomes, specifically in the X chromosome. BGN is a small leucine-rich proteoglycan acting in its soluble form as an extracellular matrix-derived danger signal. It has recently been shown that BGN deficiency in the male BALB/cA mouse strain led to sudden death due to aortic rupture, suggesting the potential role of BGN in the maintenance of the structural integrity of the aortic wall. Furthermore, BGN is reduced in individuals with Turner syndrome—a condition that affects only females, where one of the X chromosomes is missing or partially missing—who suffer from vascular anomalies including aortic dissection and rupture [41].

A sex disparity in the response to stress has been reported in different species and cell types [42–46], and the tendency of male ECs to easier undergo apoptosis in response to serum starvation fully agrees with these results. Overall, it has been suggested that male and female cells adopt different strategies to face a cellular stress induced by the same injury, with male cells more prone to apoptosis and female cells predisposed to autophagy [42–46]. The different responses to cellular stress, as well as most of the phenotypic differences between male and female cells and organisms, have been related to the sex-biased expression of genes due to their transcriptional and post-transcriptional regulation [47–49]. In humans and other mammals, microRNAs (miRNAs) are the key players in the post-transcriptional repression of mRNA targets, and evidence is accumulating for a sex-biased expression of these small regulatory RNAs [47]. To date, few miRNAs have been reported to be present in the Y chromosome whereas the X chromosome contains about 10% of the total miRNAs. Recently, the sex-dependent expression of the miR548am-5p has been proposed to control the propensity of human male dermal fibroblasts to apoptosis through its ability to alter the relative expression of pro- and anti-apoptotic proteins [45], thus confirming the possible involvement of miRNAs in the regulation of sex-relative properties. In addition, the ability of female ECs to maintain higher levels of metabolites and a better energy balance under stressed conditions in comparison with male ECs has recently been described [18]. Accordingly, the secretome from stressed female ECs contains greater amounts of proteins involved in specific metabolic pathways. Since a central role for metabolism in the phenotypic plasticity characterizing ECs has emerged in recent years [50], the recruitment of sex-specific metabolic pathways in the cellular response to stress cannot be excluded and deserves further studies.

However, although the regulation of transcription is crucial in defining the specific expression of genes in cells and tissues, it has been shown that most of the tissue-enriched transcriptome codes for secretory proteins—classified as proteins having a signal peptide, but lacking a trans-membrane region—and that secretome holds the largest fraction of tissue-specific proteome [51]. In humans, about 2600 genes—corresponding to approximately 13% of all protein-coding genes—code for potentially secreted proteins, and around 500 of these proteins were annotated as secreted in the proximity to the cell of origin, including proteins expressed in male/female tissues [52]. Thus, it is possible to hypothesize that specific regulatory programs exert a fine-tune control on the delivery of functional secretory proteins that in turn may be involved in the onset and maintenance of sex-specific cellular properties. Our results, showing different secretory phenotypes in serum-deprived male and female ECs, support the idea that the production of different sets of proteins might take part in the endothelial sex-biased response to cellular stress.

Very recently, it has been discovered that metabolites in the secretome of apoptotic cells are endowed with multiple biological functions, and not simply derived from the passive emptying of dying cells [53]. Some of these metabolites may modulate inflammation and wound healing by inducing specific gene programs in healthy neighboring cells. Other metabolites may be involved as find-me or eat-me signals in the resolution of apoptosis under which damaged cells are recruited and removed by macrophages via efferocytosis. In this scenario, our finding of elevated PTX3 levels in the secretome of male apoptotic ECs is suggestive of a still unknown role for secreted PTX3 in the establishment of innate sex-dependent properties, e.g., the response to environmental stress in human ECs. Importantly, the increased secretion of PTX3 is closely associated with the apoptotic male phenotype since PTX3 is equally expressed in male and female ECs, and no difference in the secreted quantity has been observed when ECs are not exposed to cellular stressors. About that, it is critical to remark that PTX3 is a cognate molecule of the C-reactive protein (CRP), a prototypic humoral acute phase protein helpful in the clinic as a systemic biomarker to monitor infections and inflammatory diseases [24, 37]. Similarly, PTX3 blood levels rapidly rise either in humans or mice in pathological conditions with inflammatory and/or infectious origins, and typically increase faster than CRP, very likely because of its local production. Indeed, the human PTX3 protein—a 381-amino acid glycoprotein with a 17-amino acid signal peptide for secretion—can be locally produced by various cell types, including vascular ECs, in response to pro-inflammatory cytokines or microbial moieties, thus acting as a fluid phase pattern recognition receptor able to sense cell and tissue damage and to orchestrate the outcome of the inflammatory response [37]. Hence, it is conceivable that the baseline secretion of PTX3 does not differ between unstressed ECs to precisely increase in response to cellular suffering, e.g., serum deprivation in our in vitro model. In addition, PTX3 has been involved in the regulation of vascular integrity and cardiovascular biology, although contrasting results have been so far provided either in preclinical or clinical research [35, 54, 55]. Finally, the ability to regulate recognition and clearance of apoptotic cells and debris, namely a role as eat-me signal, has been proposed for PTX3 in different cells and tissues [40, 56–59]. Our findings, showing that the silencing of PTX3 impaired efferocytosis only in male ECs, but not in females, suggest that this protein might act as an endothelial eat-me signal in a sex-dependent manner. At variance with other forms of cell death, apoptosis is a non-inflammatory process, and the timely phagocytosis of dying cells by macrophages prevents the release of inflammatory factors, the establishing of inflammation, and the development of chronic inflammatory disorders, such as atherosclerosis [38, 39, 60]. Therefore, the involvement of PTX3 in the resolution of the male apoptotic process may reflect the effort of secreted PTX3 to maintain vascular integrity and to counteract chronic endothelial inflammation via the prompt execution of the efferocytotic process.

Besides PTX3, our study demonstrated that other molecules with anti-inflammatory properties are present in the male EC secretome. Specifically, it also contains significant levels of calreticulin, one of the most characterized eat-me signals [61, 62]. In accordance with our data in PTX3-silenced male ECs, decreased levels of calreticulin have been associated with an impaired efferocytosis [63]. Another noteworthy component of apoptotic male EC secretome is annexin I, which has been actively involved in efferocytosis, in the resolution of inflammation, and in the delay of atherosclerotic plaque progression [64–66]. Moreover, it cannot be excluded that these or other secreted molecules may be responsible for further modulatory actions in nearby cells due to transcriptional/post-transcriptional mechanisms and/or metabolic reprogramming.

At variance with apoptotic male ECs, the female EC secretome contains lower quantities of PTX3, calreticulin, and annexin I. In addition, PTX3 silencing left unaffected efferocytosis in female ECs, confirming that the increased secretion of the protein, and its putative role as eat-me signal, is closely associated with the apoptotic male phenotype. However, these results do not exclude the capability of female ECs to contrast inflammation but perhaps suggest that female cells may temper endothelial inflammation through other mechanisms. As discussed above, male and female cells adopt distinct plans in response to the same cellular stressor [42–46]. Specifically, female cells appear more prone to autophagy, and our preliminary results in serum-deprived female ECs support this hypothesis (Cattaneo et al., manuscript in preparation). Since a protective role against atherosclerosis has been suggested for autophagy [67], it will be of great interest to study whether this mechanism might represent the path chosen by female ECs to contrast endothelial injures and inflammation.

### Perspective and significance

In sum, the finding of different secretory phenotypes in stressed male and female ECs advise a central role for secretory pathways and secreted proteins in the control of sex-specific cellular properties and homeostasis, thus unveiling a novel mechanism that may be responsible for sex-biased pathophysiological responses. We identified secreted PTX3 as a crucial player in the male-specific endothelial response to an apoptotic trigger, especially in the execution of efferocytosis. Although still debated, the pleiotropic functions of PTX3 in cardiovascular biology are of growing interest. Our results suggest that a fine-tuning in time and space might be responsible for the sex-dependent production/activity of PTX3 in response to environmental stressors. Overall, understanding in what manner PTX3 secretion is regulated in male and female ECs, and in what way it may balance pro- and anti-inflammatory signals at the vascular bed, might be crucial to identify novel sex-specific pathogenetic mechanisms and pharmacological targets for the prevention and treatment of endothelial dysfunction at the onset of atherosclerosis and cardiovascular disease.

### Limitations of the study

The main limitation of our study is the use of ECs from a unique source, the human umbilical vein. Actually, in recent years, organ specificity and function of the endothelium and ECs have been clearly demonstrated [68]. From this perspective, HUVECs derive from a rather unique tissue with distinctive properties (it is, among others, the first interface fetus/mother). Despite this unicity, HUVECs still represent the main model for studying in vitro properties of endothelium and ECs [69]. In fact, HUVECs undergo in vitro angiogenesis, produce NO, and respond to shear stress, hyperglycemia, and inflammatory stimuli, thus recapitulating human disease pathophysiology. In addition, sex-dependent differences in the gene expression and functional properties have been shown in male and female HUVECs by us and other groups [12–14, 17, 18, 70], indicating HUVECs as a suitable model for studying cellular biological sex and increasing the translational value of basic research. Nevertheless, whether and through which mechanisms HUVEC dimorphic properties are lifelong conserved and regulated in adult male and female ECs still need to be deeper investigated. However, a transcriptome analysis comparing HUVECs to adult human ECs has very recently shown that more than 250 genes are concordantly differentially expressed between sexes at birth and in adults [70], thus further supporting the use of HUVECs as a valuable model for basic research on the endothelium.

In addition, in our past and present studies involving HUVECs, we prepared cells from anonymized umbilical cords collected at one of the biggest obstetrics and gynecology public hospital in Milano. The cords were from healthy male or female babies born at term from healthy mothers who were drug- and infection-free. Thus, our cells offer a snapshot of the whole mother/newborn population (without any bias due to stratification or selection). In our opinion, this method of cord collection limits the enrichment of specific phenotypes and make our data even more robust. However, it should be interesting in the future to stratify the mother population—by age or cardiovascular risk, for example—to detect whether and how these factors may affect HUVEC response to stressors and/or secretome composition.

Another crucial issue regards the possibility of comparing male and female ECs obtained from dizygotic twin cords to limit genetic and environmental differences. We have already taken advantage of this approach in our previous paper to confirm the increased eNOS expression in female ECs [14]. However, the collection of twin cords is limited by a number of criticisms: (i) the incidence of spontaneous dizygotic twinning is quite low, around 1–2%; (ii) the rate of preterm labor among twins is around 60%; (iii) remarkably, HUVECs from premature cords show impaired properties when compared to cells from term cords [71]; (iv) intrauterine growth restriction is increased in twin pregnancies [72]; (v) about 75% of twins being delivered by cesarean [73]; and (vi) when childbirth is vaginal, a correlation between twin-to-twin delivery intervals and pH in umbilical arterial blood gas has been found, thus alerting about metabolic acidosis [74]. In addition, it should be considered that some differences may exist between the prenatal environment of male and female dizygotic twins. Specifically, exposure to androgens, notably testosterone, may be particularly high for the female twin in opposite-sex twin pairs, also in comparison with the female in monozygotic twins or unrelated newborns. Studies in rodents have shown that chromosomal female fetuses that are in proximity to male littermates are passively exposed to testosterone and exhibit masculinized morphological, endocrine, and behavioral phenotypes [75]. Similarly, human females exposed prenatally to a male co-twin experience in utero testosterone transfer that induces cognitive and behavioral changes [76]. Indeed, in the human embryo, testosterone directly impacts the developing brain structures and starts to be produced in male testes 6 weeks after conception [77]. The ability of testosterone to de-feminize and masculinize gene expression in a XX background is mediated by epigenetic mechanisms, and finally influences gene expression [78]. In general, studies in twins help to discover and/or amplify less pronounced sex differences due to the greatest statistical power of the paired design. At variance, sex differences identified in unrelated newborns may be robust and prevalent.

## Supplementary Information

**Additional file 1:.** Supplementary Table 1.

**Additional file 2:.** Supplementary Table 2.

**Additional file 3:.** Supplementary Figure 1.

**Additional file 4:.** Unedited western blots.

## Data Availability

The mass spectrometry proteomics data have been deposited to the ProteomeXchange Consortium via the PRIDE partner repository with the dataset identifiers PXD020375 and 10.6019/PXD020375.
