# Supplementary material for: Sex-dependent differences in the secretome of human endothelial cells
Source: Biol Sex Differ. 2021 Jan 7;12:7. doi: 10.1186/s13293-020-00350-3 (PMC7791663; doi:10.1186/s13293-020-00350-3)
Supplement: Supplementary file 1 — Additional file 1:. Supplementary Table 1. [file 13293_2020_350_MOESM1_ESM.pdf]

Supplementary Table 1. Functional enrichments of Biological Processes in the network created by STRING with proteins more secreted by male ECs.

GO-term	description	count in gene set	false discovery rate
GO:0034975	protein folding in endoplasmic reticulum	4 of 14	3,23E-06
GO:0036500	ATF6-mediated unfolded protein response	3 of 9	0,00013
GO:0019538	protein metabolic process	15 of 4194	0,00021
GO:0050896	response to stimulus	18 of 7824	0,0017
GO:0051208	sequestering of calcium ion	2 of 4	0,0029
GO:0006986	response to unfolded protein	4 of 153	0,0029
GO:0006950	response to stress	12 of 3267	0,0029
GO:0052405	negative regulation by host of symbiont molecular function	2 of 6	0,0035
GO:0042221	response to chemical	13 of 4153	0,0035
GO:0035821	modification of morphology or physiology of other organism	4 of 182	0,0035
GO:0010033	response to organic substance	11 of 2815	0,0035
GO:0031340	positive regulation of vesicle fusion	2 of 8	0,0037
GO:0051851	modification by host of symbiont morphology or physiology	3 of 71	0,0038
GO:0044092	negative regulation of molecular function	7 of 1119	0,0046
GO:0034976	response to endoplasmic reticulum stress	4 of 240	0,0053
GO:0010035	response to inorganic substance	5 of 491	0,006
GO:0071310	cellular response to organic substance	9 of 2219	0,0079
GO:0016192	vesicle-mediated transport	8 of 1699	0,0079
GO:0071345	cellular response to cytokine stimulus	6 of 953	0,0112
GO:0045807	positive regulation of endocytosis	3 of 129	0,0112
GO:0044267	cellular protein metabolic process	11 of 3603	0,0112
GO:0032940	secretion by cell	6 of 959	0,0112
GO:0009719	response to endogenous stimulus	7 of 1353	0,0112
GO:0002478	antigen processing and presentation of exogenous peptide antigen	3 of 123	0,0112
GO:0002376	immune system process	9 of 2370	0,0112
GO:0002479	antigen processing and presentation of exogenous peptide antigen via MHC class I, TAP-dependent	2 of 25	0,0113
GO:0019221	cytokine-mediated signaling pathway	5 of 655	0,0137
GO:0051235	maintenance of location	3 of 150	0,0142
GO:0042981	regulation of apoptotic process	7 of 1501	0,0142
GO:0009628	response to abiotic stimulus	6 of 1052	0,0142
GO:0045055	regulated exocytosis	5 of 691	0,0147
GO:0071495	cellular response to endogenous stimulus	6 of 1106	0,0164
GO:0044419	interspecies interaction between organisms	5 of 724	0,0168
GO:0051130	positive regulation of cellular component organization	6 of 1128	0,0172
GO:0043901	negative regulation of multi-organism process	3 of 173	0,0172
GO:0030574	collagen catabolic process	2 of 37	0,0172
GO:0050790	regulation of catalytic activity	8 of 2249	0,023
GO:0050766	positive regulation of phagocytosis	2 of 47	0,0238

GO:0043086	negative regulation of catalytic activity	5 of 809	0,0238
GO:0060627	regulation of vesicle-mediated transport	4 of 480	0,0266
GO:0050819	negative regulation of coagulation	2 of 51	0,0266
GO:0032963	collagen metabolic process	2 of 54	0,0293
GO:0071407	cellular response to organic cyclic compound	4 of 505	0,0307
GO:0014070	response to organic cyclic compound	5 of 873	0,0307
GO:0006897	endocytosis	4 of 510	0,0307
GO:0051050	positive regulation of transport	5 of 892	0,0321
GO:0048523	negative regulation of cellular process	11 of 4454	0,0321
GO:0042493	response to drug	5 of 900	0,0321
GO:0018149	peptide cross-linking	2 of 59	0,0321
GO:0030433	ubiquitin-dependent ERAD pathway	2 of 65	0,0369
GO:1901565	organonitrogen compound catabolic process	5 of 958	0,0408
GO:0009057	macromolecule catabolic process	5 of 970	0,042
GO:0048585	negative regulation of response to stimulus	6 of 1483	0,0481
