# Supplementary material for: Sex-dependent differences in the secretome of human endothelial cells
Source: Biol Sex Differ. 2021 Jan 7;12:7. doi: 10.1186/s13293-020-00350-3 (PMC7791663; doi:10.1186/s13293-020-00350-3)
Supplement: Supplementary file 2 — Additional file 2:. Supplementary Table 2. [file 13293_2020_350_MOESM2_ESM.pdf]

Supplementary Table 2. Functional enrichments of Biological Processes in the network created by STRING with proteins more secreted by female ECs.

GO-term	description	count in gene set	false discovery rate
GO:0019682	glyceraldehyde-3-phosphate metabolic process	2 of 19	0.0013
GO:0019637	organophosphate metabolic process	3 of 1011	0.0118
GO:0046496	nicotinamide nucleotide metabolic process	2 of 107	0.0118
GO:1901135	carbohydrate derivative metabolic process	3 of 1083	0.0118
GO:0005996	monosaccharide metabolic process	2 of 198	0.0132
GO:0044281	small molecule metabolic process	3 of 1779	0.0266
GO:0006796	phosphate-containing compound metabolic process	3 of 2065	0.0378
GO:0019693	ribose phosphate metabolic process	2 of 455	0.0442
