# Supplementary material for: Sex-dependent differences in the secretome of human endothelial cells
Source: Biol Sex Differ. 2021 Jan 7;12:7. doi: 10.1186/s13293-020-00350-3 (PMC7791663; doi:10.1186/s13293-020-00350-3)
Supplement: Supplementary file 3 — Additional file 3:. Supplementary Figure 1. [file 13293_2020_350_MOESM3_ESM.pdf]

Sex-dependent differences in the secretome of human endothelial cells

Maria Grazia Cattaneo[1, #, *], Cristina Banfi[2, #], Maura Brioschi[2], Donatella Lattuada[1] and Lucia M. Vicentini[1]

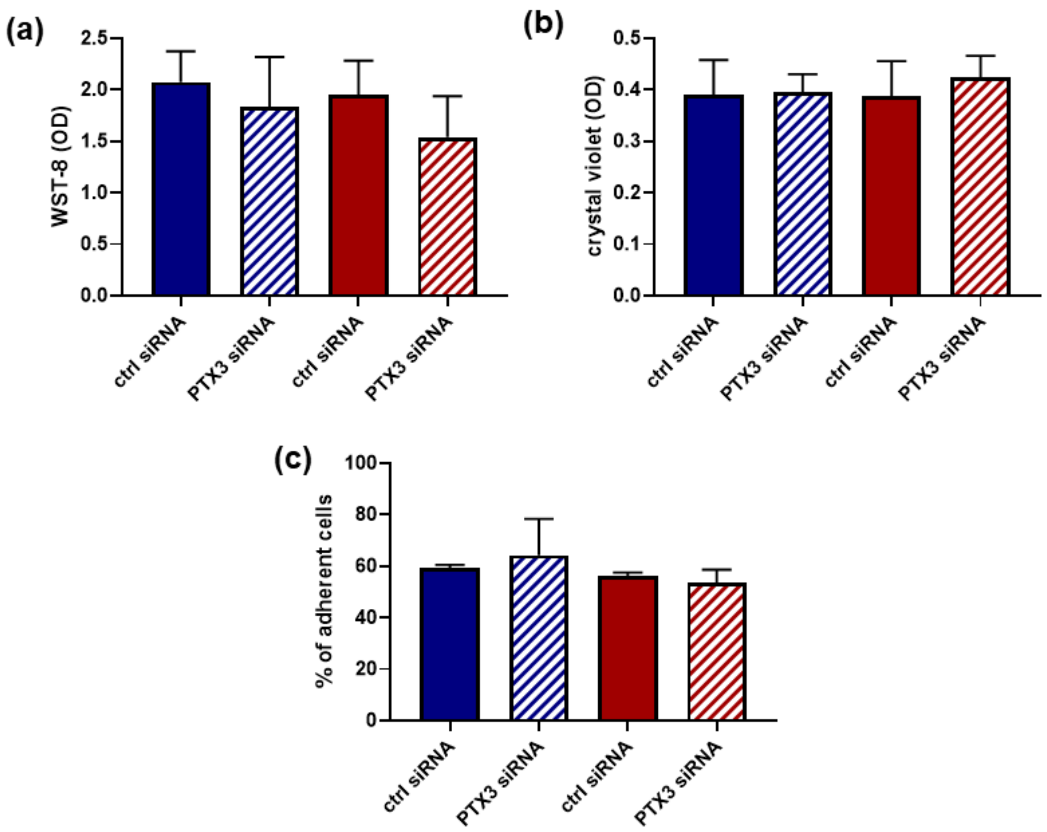

Supplementary Figure 1. Metabolic activity (**a**) and cell number (**b**) were evaluated 48h after transfection with control (ctrl, solid bars) or PTX3 siRNA (diagonal bars) in male and female ECs (blue and deep red bars, respectively) cultured in 20% FBS. Data are expressed as WST-8 or crystal violet absorbance (OD) and are the mean ± s.e.m. of 3 independent experiments. In (**c**), calcein-loaded ECs were incubated overnight in 2% FBS, and then plated on 0.1% gelatin-coated wells. After 2 h, non-adherent cells were removed by washing and the well-associated fluorescence was measured. Data are expressed as percent (%) of the total added fluorescence and are the mean ± s.e.m. of 2 independent experiments.

Supplementary methods

Metabolic activity was evaluated through the ability of viable male and female ECs of bio-reducing WST-8 to an orange formazan product by means of cellular dehydrogenases. WST-8 was added for the last 3 h of incubation according to the manufacturer's instructions (Dojindo). Absorbance was measured at 450 nm using a microplate reader.
