# Supplementary material for: Sex-dependent differences in the secretome of human endothelial cells
Source: Biol Sex Differ. 2021 Jan 7;12:7. doi: 10.1186/s13293-020-00350-3 (PMC7791663; doi:10.1186/s13293-020-00350-3)
Supplement: Supplementary file 4 — Additional file 4:. Unedited western blots. [file 13293_2020_350_MOESM4_ESM.pdf]

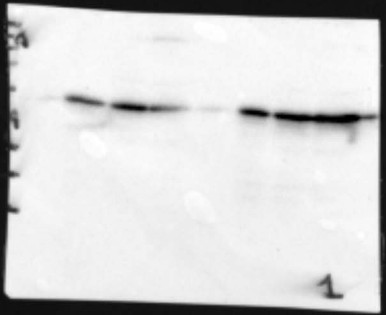

Lanes 2/3 and 6/7 (from the right) have been cropped for fig. 4C (procaspase 3). Exposure time 1.0 sec.

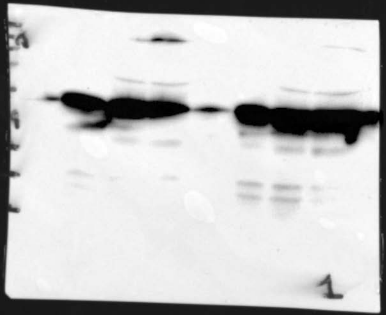

Lanes 2/3 and 6/7 (from the right) have been cropped for fig. 4C (cleaved caspase 3). Exposure time 53.8 sec.

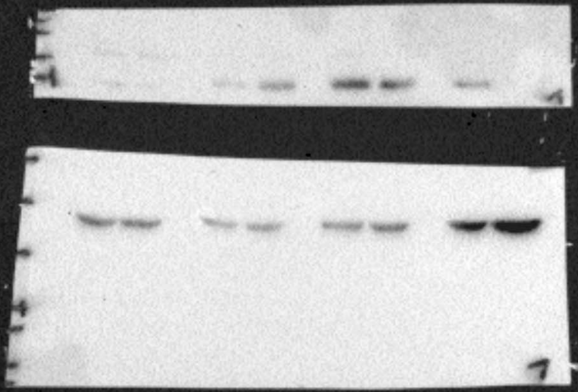

Lanes 5 and 6 (from the right) have been cropped for fig. 4D.
Upper bands, PARP-1; lower bands, actin.
Exposure time 124.7 sec
